# Equipping the next generation of clinicians for addressing conflict mental health: A role for Geopsychiatry

**DOI:** 10.1371/journal.pmen.0000094

**Published:** 2024-08-02

**Authors:** Joseph El-Khoury, Audrey McMahon, Fatema Kazem, Mia Atoui, Joao Mauricio Castaldelli-Maia, Joana Corrêa de Magalhães Narvaez, Julio Torales, Myrna Lashley, Mike Campbell, Michael Liebrenz, Alexander Smith, Yunyu Xiao, Albert Persaud

**Affiliations:** 1 Department of Psychiatry United Arab Emirates University, Al Ain, United Arab Emirates; 2 Doctors Without Borders, Montreal, Canada; 3 Kuwait University, Kuwait City, Kuwait; 4 Embrace, Beirut, Lebanon; 5 Department of Psychiatry, University of São Paulo, Sao Paulo, Brazil; 6 Universidade Federal de Ciências da Saúde de Porto Alegre (UFCSPA), Porto Alegre, Brazil; 7 Department of Medical Psychology, National University of Asunción, School of Medical Sciences, San Lorenzo, Paraguay; 8 Division of Trans-Cultural Psychiatry, Department of Medicine, McGill University, Montreal, Canada; 9 Faculty of Medical Sciences, University of the West Indies, Cave Hill, Barbados; 10 Department of Forensic Psychiatry, University of Bern, Bern, Switzerland; 11 New York-Presbyterian Department of Population Health Sciences Department of Psychiatry, Weill Cornell Medicine, New York, New York, United States of America; 12 Institute of Psychiatry, Psychology & Neuroscience: Kings College, London, United Kingdom; PLOS: Public Library of Science, UNITED KINGDOM OF GREAT BRITAIN AND NORTHERN IRELAND

The past two decades have seen a surge in violent conflicts worldwide, leading to 238,000 conflict-related fatalities in 2022 alone [1]. This challenges the perception that conflict is limited to ‘unstable’ regions, highlighting its global reach. Active wars in Ukraine, Sudan and Palestine have drawn attention onto conflicts even further. Beyond immediate death tolls, the enduring psychosocial impacts are profound, with conditions like Post Traumatic Stress Disorder (PTSD) affecting millions [2, 3]. During its last World Health Assembly, the World Health Organization approved a crucial resolution to integrate mental health and psychosocial support (MHPSS) across all stages of emergencies, including conflicts, disasters, and humanitarian crises. The distant and recent experience from conflict zones raises doubt on whether local healthcare systems and external interventions have the needed skills and experience to deliver in times of crisis [4].

## Current gaps in training

Psychiatric training often lacks comprehensive education on conflict-related mental health. A US survey revealed only 20% of psychology doctoral programs offer trauma-focused training [5]. Similarly, a Canadian study found significant gaps in preparing residents to handle war-induced trauma [6]. Despite some programs like George Washington University’s global mental health (GMH) initiative, which includes rotations in conflict zones, these are exceptions rather than the norm [7].

The lack of training means that many clinicians are ill-prepared to deal with the complex mental health needs of populations affected by conflict. For example, traditional psychiatric training often focuses on PTSD without addressing other common conflict-related conditions such as depression, anxiety, and substance abuse. Furthermore, training programs rarely include components on cultural competence, which is crucial for effectively treating refugees and migrants who may have different expressions of psychological distress.

## From textbooks to conflict zones

The skills needed to work in conflict zones surpass psychiatric knowledge and competence and necessitate to go beyond one’s epistemological foundation. It calls for the psychiatrist to explore the intersection of global mental health, transcultural psychiatry, social sciences, politics and geography. During war, mental health professionals are compelled to look beyond the individual as to avoid the risk of depoliticizing suffering and of pathologizing appropriate responses in the face of trauma and collective violence.

A fundamental aspect of preparing residents to practicing psychiatry in conflict and humanitarian zones, and with refugee populations in host countries, resides in the imperative learning of cultural humility and cultural safety. The concept of cultural competence was developed first and has aimed to increase cultural sensibility and the provision of care to culturally diverse population. However, it mainly focuses on developing “competence” through knowledge to increase the self-confidence of the physician without necessarily ensuring cultural humility and safety, both crucial to offering care that is person-centered and that truly engages the lifeworld of the other [8].

Furthermore, the notion of PTSD and trauma as taught by Western psychiatry seems to not encompass the spectrum of traumatic symptomatology observed in conflict zones and the significant impact they have on populations. In many contexts torn by armed conflicts, there is no post-trauma: many conflicts are protracted, ongoing and linger in all layers of daily life. As seen previously, trauma that is intentionally inflicted by another can impact in ways that alter one’s system of meanings and worldview. In the same way, migration, forced displacement, and the targeting of ethnicity when it comes to political violence may leave profound psychological scars that are not fully portrayed by PTSD. The understanding of trauma and the practicality of psychiatric consultation when working with populations affected by war demands a cultural and social systemic perspective and a broadening of the concept of trauma [9, 10].

Lastly, another important consideration in preparing mental health professionals to clinical practice in conflict zones concerns the prevention and management of vicarious traumatization and how to care for oneself when experiencing contexts that inadvertently shake one’s perception of providing healthcare, and sometimes of humanity as whole. Trainees seem to be massively unprepared for this.

## A public health necessity

The psychosocial scars of conflict may vary in manifestation across contexts and cultures, yet their impact is universal and transgenerational. They infiltrate the very fabric of societies, haunting collective memories and presenting critical challenges for psychiatrists and psychologists across the globe.

Training programs must adapt to geopolitical and cultural contexts. Social determinants of health, including discrimination and social exclusion, adversely affect migrant mental health [11, 12]. Refugees face higher rates of mental health conditions, exacerbated by post-migration stressors [11, 13]. Addressing these challenges requires integrating cultural competence and trauma-informed care into clinician training.

Social determinants of health play a significant role in the mental health outcomes of conflict-affected populations. These include access to safe environments, adequate food, housing, healthcare, and employment. Interpersonal factors such as experiences of social exclusion, discrimination, low social status, gender inequalities, and exclusion from social and health services also impact mental health [14]. Training programs must incorporate these elements to prepare clinicians for the realities of treating conflict-affected populations.

## The role of geopsychiatry

Geopsychiatry offers a crucial framework for understanding and addressing the mental health impacts of conflict. It examines how external forces like wars impact local mental health, emphasizing the need for interdisciplinary collaboration [15, 16]. Effective responses require cooperation among psychiatrists, policymakers, and human rights experts. However, research in geopsychiatry is still emerging, necessitating further study and integration into training programs.

By examining the influence of geopolitical factors on mental health, geopsychiatry underscores the importance of addressing the root causes of conflict-related mental health issues. This approach is not only about treating symptoms but also about understanding and mitigating the broader societal impacts of conflict. For instance, the ongoing conflict in Ukraine has shown that psychological trauma extends beyond immediate survivors to entire communities, requiring a holistic response that includes mental health support, community rebuilding, and policy changes. Similarly, the war in Palestine has had social and psychological repercussions well beyond the battleground impacting communities around the world, with social media playing an important role in secondary traumatization.

## Policy recommendations

1. **Integrate Geopsychiatry into Training Curricula:** Mental health training programs should include geopsychiatry to prepare clinicians for the unique challenges of conflict-related mental health. This integration would help clinicians understand the broader geopolitical factors influencing mental health and develop more effective treatment strategies.
2. **Expand Global Mental Health Programs:** Universities should expand GMH programs to include more comprehensive training on conflict-related mental health issues. This could include optional rotations targeting conflict zones, as well as training in trauma-informed care.
3. **Increase Funding for Research:** More funding is needed for research on geopsychiatry to develop evidence-based practices for treating conflict-related mental health issues. This research should focus on the long-term impacts of conflict and the effectiveness of different treatment approaches.
4. **Develop Standardized Guidelines:** National and international health organizations should develop standardized guidelines for training clinicians in conflict-related mental health. These guidelines should include specific competencies on trauma-informed care, cultural competence, and the social determinants of health.
5. **Improve cultural and contextual competency:** Emphasize the importance of having teachers from different backgrounds, from theory to practice, but also professors from the “Global South” with firsthand experiences of being from and working in conflict zones.
6. **Leverage technology:** The growth of telehealth competency in the field of mental health allows for clinicians to assess, diagnose and treat without placing themselves in the way of harm in active conflict zones.

## Conclusion

To address the escalating global conflicts and their profound psychosocial impacts, it is essential to integrate geopsychiatry into mental health training programs. This emerging field provides critical insights into the interplay between geographic and psychiatric factors, crucial for developing culturally sensitive and effective interventions. By equipping clinicians with the knowledge and skills to address conflict-related mental health issues, we can foster resilience and healing in affected populations, ultimately contributing to more stable and peaceful societies. Psychiatrists, psychologists and other mental health professionals should be at the forefront of this effort.
